# Elements Human Psysiology

**Published:** 1893-07

**Authors:** 


ELEMENTS OF HUMAN PHYSIOLOGY.—By Ernest H. Sterling, M. D., London, M. R. C. P., Joint Lecturer on Physiology at Guy's Hospital, London, member of Physiological Society, etc. 100 illustrations. 12 mo., 436 pages. Cloth, $2. P. Blackiston, Son & Co., Publishers, 1012 Walnut st., Philadelphia.

This is a splendidly written little text book, in which the author has presented the main facts of Physiology that are of interest to the medical student, in a clear and concise manner. The book gives the latest physiological experiments and investigations, and thus possesses the special merit of being fully up to date.

When we consider how rapidly the science of physiology is developing, due to the experiments and investigations of leading physiologists of the present day. this feature of the book can be fully appreciated.

The subjects treated of are presented in such a clear and concise manner, that no student need have any difficulty in fully understanding them. The author very properly leaves the subject of histology largely to works on anatomy. The introductory chapter is within itself an epitome of physiology, as understood and taught at the present time. The chapters on the chemical constituents of the body, blood and lymph; the general properties of nerve fibres; the vascular mechanism; digestion, perspiration, excretion, etc., are especially fine. The author says much in few words, and these few words are plain and to the point.

The book is a very meritorious one and should have a large sale. H.

